# Nrf2 promotes breast cancer cell migration via up‐regulation of G6PD/HIF‐1α/Notch1 axis

**DOI:** 10.1111/jcmm.14241

**Published:** 2019-02-26

**Authors:** Hong‐Sheng Zhang, Zhong‐Guo Zhang, Guang‐Yuan Du, Hong‐Liang Sun, Hui‐Yun Liu, Zhen Zhou, Xiao‐Meng Gou, Xi‐Hao Wu, Xiao‐Ying Yu, Ying‐Hui Huang

**Affiliations:** ^1^ College of Life Science & Bioengineering, Beijing University of Technology Beijing China

**Keywords:** breast cancer, G6PD, HIF‐1α, Notch1, Nrf2

## Abstract

Abnormal metabolism of tumour cells is closely related to the occurrence and development of breast cancer, during which the expression of NF‐E2‐related factor 2 (Nrf2) is of great significance. Metastatic breast cancer is one of the most common causes of cancer death worldwide; however, the molecular mechanism underlying breast cancer metastasis remains unknown. In this study, we found that the overexpression of Nrf2 promoted proliferation and migration of breast cancers cells. Inhibition of Nrf2 and overexpression of Kelch‐like ECH‐associated protein 1 (Keap1) reduced the expression of glucose‐6‐phosphate dehydrogenase (G6PD) and transketolase of pentose phosphate pathway, and overexpression of Nrf2 and knockdown of Keap1 had opposite effects. Our results further showed that the overexpression of Nrf2 promoted the expression of G6PD and Hypoxia‐inducing factor 1α (HIF‐1α) in MCF‐7 and MDA‐MB‐231 cells. Overexpression of Nrf2 up‐regulated the expression of Notch1 via G6PD/HIF‐1α pathway. Notch signalling pathway affected the proliferation of breast cancer by affecting its downstream gene HES‐1, and regulated the migration of breast cancer cells by affecting the expression of EMT pathway. The results suggest that Nrf2 is a potential molecular target for the treatment of breast cancer and targeting Notch1 signalling pathway may provide a promising strategy for the treatment of Nrf2‐driven breast cancer metastasis.

## 1 INTRODUCTION

Breast cancer is a complex, heterogeneous disease and one of the most common female cancers worldwide.^1^ Although great progress has been achieved in early diagnosis and systemic therapy of breast cancer in recent years, metastasis remains a major obstacle in the effective treatment of breast cancer. In breast cancer, the role of NF‐E2‐related factor 2 (Nrf2) in tumour growth is controversial and likely context dependent.^2^, ^3^ However, emerging evidence has indicated that increased activity can enhance the metastatic potential of breast cancer cells. Understanding the molecular mechanisms underlying breast cancer metastasis is a key to develop novel therapeutic approaches to treat metastatic breast cancer.

Redox status is a well‐recognized actor in the adaptation of cancer cells to therapy. Redox adaptation is important in cancer cells drug resistance. The transcription factor Nrf2 is the master regulator of antioxidant and cytoprotective systems.^4^ The antioxidant response is principally mediated by the transcription factor Nrf2, which induces the transcriptional activation of several genes involved in glutathione (GSH) synthesis, chemoresistance and cytoprotection.^5^ In recent years, accumulating evidence implies the importance of Nrf2 deregulation in tumourigenesis.^5^ Despite the recent progress in the characterization of Nrf2 transcription factors, biological functions of Nrf2 remain to be explored. Several types of cancer cells display a large amount of reactive oxygen species (ROS), due to an aberrant metabolism, mitochondrial dysfunction or activation of oncogenes.^6^ Under physiological conditions, Nrf2 localizes in the cytoplasm where it is bound by Kelch‐like ECH‐associated protein 1 (Keap1). Kelch‐like ECH‐associated protein 1 forms a complex with Cul3 and Rbx1, and this E3 ubiquitin ligase complex can bind and ubiquitinate Nrf2, resulting in Nrf2 proteasomal degradation.^7^ The stabilized Nrf2 accumulates in nuclei, heterodimerizes with small Maf proteins and activates target genes for cytoprotection through the antioxidant response element (ARE)/electrophile response element (EpRE).^8^ The function of Nrf2 in chemoresistance has been demonstrated in diverse types of cancers, including cisplatin resistant bladder cancers.^9^

The migration and invasion of tumour cells are crucial in cancer metastasis.^10^ Warburg effect is an important expression of tumour cell metabolic reprogramming. However, tumour metabolic reprogramming occurs in many metabolic pathways, including glycolysis, the pentose phosphate pathway (PPP) and the Krebs cycle process.^11^ The PPP is irreplaceable in the rapid proliferation of tumour cells regarding the provision of raw materials for macromolecular biosynthesis and maintenance of cellular redox status.^12^ Recent studies have suggested that PPP is raised in many tumour cells, but maintenance of high hyperplastic in tumour cells through the PPP remains unanswered. Notch signalling pathway is a classical pathway. Recent studies show that Notch pathway was involved in cell proliferation, differentiation, migration and invasion.^13^ It should be noted that Nrf2 is related to Notch pathway.^14^ The synergy of Nrf2 and Notch pathway promotes survival rate of tumour cells, differentiation, invasion and metastasis in the condition of abnormal expression of Nrf2 and Notch pathway.^15^ It is possible that Nrf2 can adjust the Notch pathway through affecting the PPP and leads to a change in breast cancer cell proliferation and migration, and need to explore its mechanism.

In this study, we demonstrate that Nrf2 leads to increased proliferation, migration, invasion in breast cancer cells. We found, for the first time, a novel function of Nrf2 in the Notch1 signalling pathway via PPP. Modulation of glucose‐6‐phosphate dehydrogenase (G6PD)/ Hypoxia inducing factor 1α (HIF‐1α) expression by Nrf2 is therefore involved in the Notch1 pathway‐mediated regulation proliferation, migration, invasion of breast cancer.

## 2 MATERIALS AND METHODS

### 2.1 Chemical reagents and antibodies

Polyclonal antibodies to Notch1, Notch2, Notch3, Notch4, Dll1, Dll3, Dll4, Jagged1, Jagged2, HIF‐1α, N‐cadherin, E‐cadherin and Snail1 were purchased from Cell Signaling Technology (Beverly, MA). Polyclonal antibodies to G6PD, transketolase (TKT), Nrf2, Keap1 and monoclonal antibody to β‐actin were purchased from Abcam (Cambridge, UK). The dual‐luciferase reporter assay kit was obtained from Promega (Madison, WI).

### 2.2 Cell culture

Human breast cancer cells (MCF‐7, MDA‐MB‐231) were grown in DMEM containing 10% foetal bovine serum (FBS) and 1% penicillin‐streptomycin solution in 5% CO_2_ at 37°C. Normal human mammary epithelial cells, MCF‐10A cells, were maintained in DMEM/F12 (Invitrogen, Carlsbad, CA) supplemented with 5% horse serum (Invitrogen, Carlsbad, CA, USA), 20 ng/mL epidermal growth factor (Sigma‐Aldrich, St. Louis, MO), 0.5 μg/mL hydrocortisone (Sigma‐Aldrich), 10 μg/mL insulin (Sigma‐Aldrich), 100 ng/mL cholera toxin (Sigma‐Aldrich) and 100 U/mL penicillin/streptomycin. HEK‐293T cells were grown in DMEM supplemented with 10% FBS and 100 U/mL penicillin/streptomycin.

### 2.3 Plasmid construction and virus packaging

The Nrf2 gene (GenBank accession No. NM_006164) and the Keap1 gene (GenBank accession No. NM_203500) were cloned into pCDH‐CMV‐MCS‐EF1‐Puro vector. Short hairpin RNA (shRNA) sequences targeting G6PD, TKT, Nrf2 or Keap1 were constructed into pLKO.1 lentiviral vectors for viral packaging. Viral transduction was performed as follows: HEK‐293T cells were cotransfected with pLKO.1 constructs and packaging plasmids. The media containing progeny virus released from HEK‐293T cells were collected and used to transduce specific cells.^16^ The cloning strategy sequences were as following: GBD‐NRF2‐F: 5′‐TGA CCT CCA TAG AAG ATT CTA GAA TGA TGG ACT TGG AGC TGC CGC‐3′; GBD‐NRF2‐R: 5′‐GCG ATC GCA GAT CCT TCG CGG CCG CCT AGT TTT TCT TAA CAT CTG GCT TCT TAC TTT TGG GAA CAA GG‐3′; GBD‐KEAP1‐F: 5′‐TGA CCT CCA TAG AAG ATT CTA GAA TGC AGC CAG ATC CCA GGC C‐3′; GBD‐KEAP1‐R: 5′‐GCG ATC GCA GAT CCT TCG CGG CCG CTC AAC AGG TAC AGT TCT GC TGG TCA AT C TG‐3′; ShNRF2‐F: 5′‐GAT CCG TAA GAA GCC AGA TGT TAA CTC GAG TTA ACA TCT GGC TTC TTA CGC TTT TTG G‐3′; ShNRF2‐R: 5′‐AAT TCC AAA AAG CGT AAG AAG CCA GAT GTT AAC TCG AGT TAA CAT CTG GCT TCT TAC G‐3′; ShKEAP1‐F: 5′‐GAT CCG AAT GAT CAC AGC AAT GAA CTC GAG TTC ATT GCT GTG ATC ATT CGC TTT TTG G‐3′; ShKEAP1‐R: 5′‐AAT TCC AAA AAG CGA ATG ATC ACA GCA ATG AAC TCG AGT TCA TTG CTG TGA TCA TTC G‐3′; ShHIF‐1α‐F: 5′‐GAT CCC GGC GAA GTA AAG AAT CTG AAC TCG AGT TCA GAT TCT TTA CTT CGC CGG CTT TTT GG‐3′; ShHIF‐1α‐R: 5′‐AAT TCC AAA AAG CCG GCG AAG TAA AGA ATC TGA ACT CGA GTT CAG ATT CTT TAC TTC GCC GG‐3′; ShG6PD‐F: 5′‐GAT CCC CGG CAA CAG ATA CAA GAA CGT GAA CTC GAG TTC ACG TTC TTG TAT CTG TTG TTT TTG G‐3′; ShG6PD‐R: 5′‐AAT TCC AAA AAC AAC AGA TAC AAG AAC GTG AAC TCG AGT TCA CGT TCT TGT ATC TGT TGC CGG G‐3′; ShTKT‐F: 5′‐GAT CCC CGG GAT GAC CAG GTG ACC GTT ATC CTC GAG GAT AAC GGT CAC CTG GTC ATC TTT TTG G‐3′; ShTKT‐R: 5′‐AAT TCC AAA AAG ATG ACC AGG TGA CCG TTA TCC TCG AGG ATA ACG GTC ACC TGG TCA TCC CGG G‐3′; ShHes1‐F: 5′‐GAT CCC CGG CTT CAG CGA GTG CAT GAG TCG AGT CAT GCA CTC GCT GAA GCC GGG CTT TTT G‐3′; ShHes‐1‐R: 5′‐AAT TCC AAA AAG CCC GGC TTC AGC GAG TGC ATG ACT CGA GTC ATG CAC TCG CTG AAG CCG G‐3′.

### 2.4 Real‐time reverse transcription‐polymerase chain reaction (RT‐PCR)

Total RNA was separated from different cells with RNAprep pure Cell Kit (Takara, Dalian, China) and reversely transcribed to cDNA for RT‐PCR. Quantitative PCR was implemented with SYBR Premix Ex Taq II (Takara, China). Amplification conditions: 95°C for 30 seconds, 40 cycles of 95°C for 10 seconds, 58°C for 10 seconds and 72°C for 10 seconds. The expression of genes was normalized to that of glyceraldehyde‐3‐phosphate dehydrogenase (GAPDH) in all samples. Relative quantification method (^∆∆^Ct) was used to calculate the change. The primer sequences were as following: GAPDH‐F: 5′‐GGC ATC CTG GGC TAC ACT GA‐3′; GAPDH‐R: 5′‐GTG GTC GTT GAG GGC AAT G‐3′; NRF2‐F: 5′‐CAT GCC CTC ACC TGC TAC TT‐3′; NRF2‐R: 5′‐GTT CTG GTG ATG CCA CAC TG‐3′; G6PD‐F: 5′‐AAG AAC GTG AAG CTC CCT GA‐3′; G6PD‐R: 5′‐AAT ATA GGG GAT GGG CTT GG‐3′; TKT‐F: 5′‐CGC TTT GTG CTC TCC AAG GGC‐3′; TKT‐R: 5′‐AAG CTT GTT TCG GGA CCG GG‐3′; NOTCH1‐F: 5′‐GTC CCA CCC ATG ACC ACT ACC CAG TTC‐3′; NOTCH1‐R: 5′‐GGG TGT TGT CCA CAG GTG A‐3′; NOTCH2‐F: 5′‐TGA CGT TGA TGA GTG TAT CTC CAA GCC‐3′; NOTCH2‐R: 5′‐GTA GCT GCC CTG AGT GTT GTG G‐3′; NOTCH3‐F: 5′‐CCG ATT CTC CTG TCG TTG TCT CC‐3′; NOTCH3‐R: 5′‐TGA ACA CAG GGC CTG CTG AC‐3′; NOTCH4‐F: 5′‐CCA GCA GAC AGA CTA CGG TGG AC‐3′; NOTCH4‐R: 5′‐GCA GCC AGC ATC AAA GGT GT‐3′; DLL1‐F: 5′‐CTC TTC CCC TTG TTC TAA C‐3′; DLL1‐R: 5′‐ACA GTC ATC CAC ATT GTC‐3′; DLL3‐F: 5′‐TCT ATC TTG TCC CTT CTC TAT CA‐3′; DLL3‐R: 5′‐AAT CAT TCA GGC TCC ATC TC‐3′; DLL4‐F: 5′‐TGA CAA GAG CTT AGG AGA G‐3′; DLL4‐R: 5′‐GCT TCT CAC TGT GTA ACC‐3′; Jagged1‐F: 5′‐TGG GAA CTG TTG TGG TGG AGT CCG‐3′; Jagged1‐R: 5′‐GTG ACG CGG GAC TGA TAC TCC T‐3′; Jagged2‐F: 5′‐AAG GTG GAA ACA GTT GT‐3′; Jagged2‐R: 5′‐CAC GGG CAC CAA CAG‐3′; HIF‐1α‐F: 5′‐AGT GTA CCC TAA CTA GCC G‐3′; HIF‐1α‐R: 5′‐CAC AAA TCA GCA CCA AGC‐3′; Hes1‐F: 5′‐AGG CGG ACA TTC TGG AAA TG‐3′; Hes1‐R: 5′‐TCG TTC ATG CAC TCG CTG A‐3′; Snail‐F: 5′‐TTC TTC TGC GCT ACT GCT GCG‐3′; Snail‐R: 5′‐AGA AGG AGA GGT ATG GAC GGG‐3′; E‐cadherin‐F: 5′‐TCC CAT CAG CTG CCC AGA AA‐3′; E‐cadherin‐R: 5′‐ATT GTC CTT GTG TCC TCA GT‐3′; N‐cadherin‐F: 5′‐GAC CAG GAC TAT GAC TTG AG‐3′; N‐cadherin‐R: 5′‐ACC ACC ACT ACT TGA GGA A‐3′.

### 2.5 Western blotting

Cells were collected with RIPA lysis buffer that comprised protease inhibitor cocktail PMSF at a final concentration of 0.1%. Cells were placed for 30 minutes on the ice and collected at 12 000 g for 20 minutes at 4°C. Bicinchoninic acid protein assay kit to detect the contents of protein. Proteins were loaded onto a SDS‐polyacrylamide gel and transferred to a nitrocellulose membrane; immunoblotting was performed with primary and secondary antibodies. Proteins were visualized with the Canon Chemiluminescence imaging system.^17^

### 2.6 Cell proliferation assay

Cell proliferation assay was completed by CellTiter 96™ AQueous One Solution Cell Proliferation Assay (MTS) Kit.^18^ Cells were inoculated at 5 × 10^3^ per well into 96‐well plates at 37°C, 5% CO_2_. Appropriate concentration of drug was added into the wells. After 12, 24, 36, 48, 60 and 72 hours, cells were added with 10 μL MTS Reagent and 90 μL medium for 1 hour at 37°C. Then absorbance of each well was tested at 490 nm using a microplate spectrophotometer.

### 2.7 Wound healing assay

Cells were cultured in six‐well plates and incubated in culture medium at 37°C in a 5% CO_2_ incubator. A wound area was made with a 200 μL pipette tip following formation of a confluent monolayer. Cells were then incubated in the same condition for 24 hours. Images were captured at 0 and 24 hours. All experiments were performed in triplicates.^16^

### 2.8 Transwell cell invasion assays

Transwell cell invasion assays were performed as previously described.^10^ In brief, an appropriate number of cells was seeded in the upper insert chamber with serum‐free medium. DMEM (500 μL) containing 10% FBS was plated at the bottom chamber. After 24 hours incubation, cells were rinsed and stained with crystal violet solution (Sigma‐Aldrich). All the results of transwell assay have been quantified by counting the invasive cell number.

### 2.9 Luciferase assay

The Notch1 promoter was cloned by PCR from genomic DNA of human MDA‐MB‐231 cells. PCR‐based cloning was used to generate a segment (−1998 to +76) of the Notch‐1 gene promoter. Then the amplified products were ligated into the pGL3 basic vector using NheI and XhoI sites, respectively, named pGL3‐Notch1. Primer sequences for Notch‐1 promoter cloning: Forward: 5′‐ACC AGC TAG CAC CCC CTA TCC AGG GAT C‐3′, Reverse: 5′‐TGG GCT CGA GCC TAC CTC GTG CGG CG‐3′. To evaluate the Notch1 signalling pathway activity, the Notch1 firefly luciferase and the thymidine kinase promoter‐Renilla luciferase (pTK‐RL) Renilla luciferase constructs were applied to measure Notch1 pathway activation. The reporter vector was transfected into MCF‐7 and MDA‐MB‐231 cells using LipofectamineTM 2000 transfection Reagent (Invitrogen) for 8 hours according to the manufacturer's protocol. The cells were lysed and luciferase activity was measured using the Dual‐Luciferase Assay System (Promega) and a BioTek Luminometer. Firefly luciferase activity was normalized to activity of Renilla Luciferase. For each experiment, the luciferase assay was performed three times.

### 2.10 Statistical analysis

ANOVA was used for three‐group comparisons; consequence was displayed as mean ± standard error of mean (mean ± SEM). A *P*‐value less than 0.05 was considered statistically significant.

## 3 RESULTS

### 3.1 Nrf2 overexpression and Keap1 knockdown promote proliferation and migration of breast cancer cells

**Figure 1:**
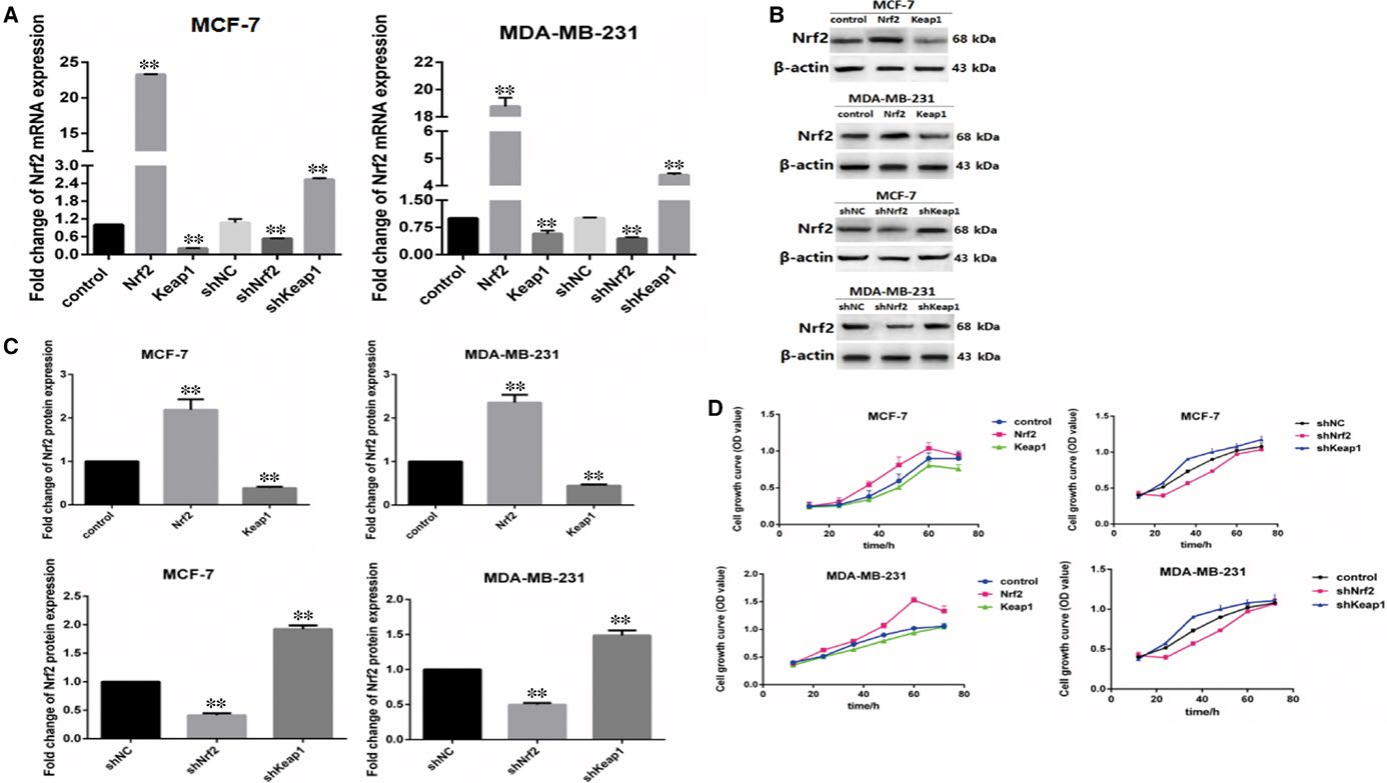
Nrf2 overexpression and Kelch‐like ECH‐associated protein 1 (Keap1) knockdown promote proliferation of breast cancers cells. A, Expression of Nrf2 mRNA in MCF‐7 and MBA‐DA‐231 cells was examined by qRT‐PCR. GADPH was used as a reference for RNA. B, Expression of Nrf2 protein in MCF‐7 and MBA‐DA‐231 cells was performed by western blotting analysis. The signal obtained with a β‐actin antibody served as loading control. Blots were the representative of three independent experiments. C, Quantitative analysis of Nrf2 protein in MCF‐7 and MBA‐DA‐231 cells was shown. D, Cell viability was analysed by MTS assay. Results were means ± SEM of three independent experiments. **P < 0.01 compared to untreated cells

To understand the potential function of Nrf2 in breast cancer progression and metastasis, Nrf2 and Keap1‐specific shRNAs and overexpression of Nrf2 and Keap1 plasmid were utilized to detect Nrf2 mRNA and protein expression with RT‐qPCR and western blotting analysis. Nrf2‐specific shRNA or overexpression of Nrf2 caused a decrease in Nrf2 mRNA and protein expression in MCF‐7 and MDA‐MB‐231 cells. In contrast, the plasmid for overexpression of Nrf2 and knockdown of Keap1 caused an increase in Nrf2 mRNA and protein in MCF‐7 and MDA‐MB‐231 cells (Figure 1A‐C).

**Figure 2:**
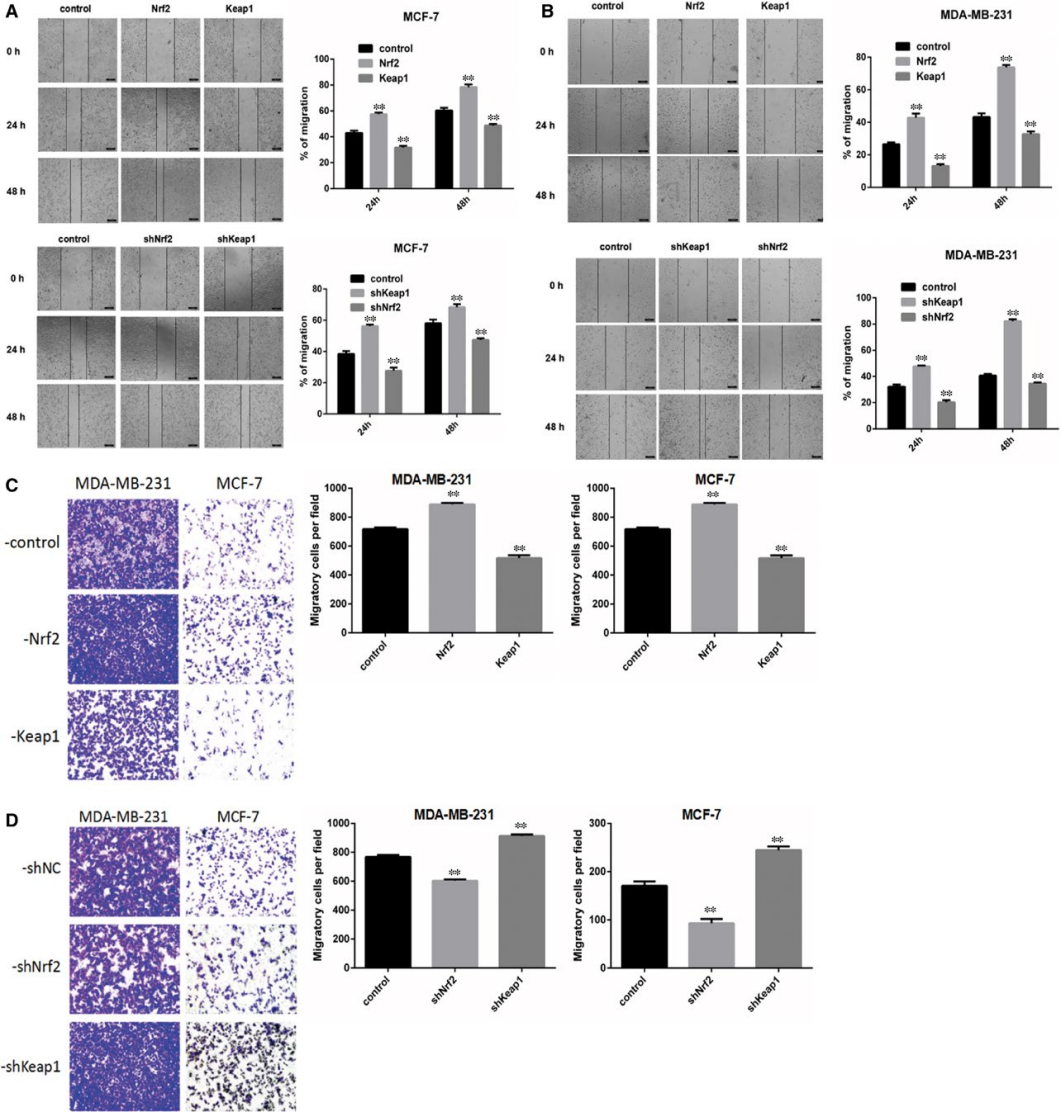
Nrf2 overexpression and Kelch‐like ECH‐associated protein 1 (Keap1) knockdown promote migration and invasion of breast cancers cells. A, Wound healing assay with MCF‐7 cells expressing shNrf2, shKeap1, Nrf2 or Keap1. B, Quantitative analysis of wound healing assay with MDA‐MB‐231 cells expressing shNrf2, shKeap1, Nrf2 or Keap1. Images were captured 24, 48, 72 h after wound was formed. The percentage of migration was assigned as 100% when complete fusion occurred, and 0% at *t* = 0 h. Relative migratory rate was shown in the graph. C, Transwell invasion assay of MCF‐7 and MDA‐MB‐231 cells expressing Nrf2 or Keap1 for 24 h. D, Quantitative analysis of transwell invasion assay of MCF‐7 and MDA‐MB‐231 cells expressing shNrf2 or shKeap1 for 24 h. Representative figures from three independent experiments were shown. Results were means ± SEM of three independent experiments. **P < 0.01 compared to untreated cells

To investigate the role of Nrf2 in breast cancer cell proliferation, we performed an MTS cell proliferation assay to establish cell growth curves for up to 96 hours. Nrf2 overexpression and Keap1 knockdown were verified to accelerate proliferation in MCF‐7 and MDA‐MB‐231 cells. Kelch‐like ECH‐associated protein 1 overexpression and Nrf2 knockdown were found to reduce proliferation in MCF‐7 and MDA‐MB‐231 cells (Figure 1D). Moreover, we performed wound healing assay and transwell assay to investigate the influence of Nrf2 on migration and invasion of it in MCF‐7 and MDA‐MB‐231 cells. In wound healing assay, the migration of cells between wound edges was significantly increased in Nrf2 overexpression and Keap1 knockdown cells compared with control cells. Conversely, Keap1 overexpression and Nrf2 knockdown in MCF‐7 and MDA‐MB‐231 cells had the opposite effect (Figure 2A,B). In the invasion assay, we observed similar results. Nrf2 overexpression and Keap1 knockdown in MCF‐7 and MDA‐MB‐231 cells had higher invasion rate than control cells; while Keap1 overexpression and Nrf2 knockdown in MCF‐7 and MDA‐MB‐231 cells had the opposite effect (Figure 2C,D). These results indicate that Nrf2 acts as a tumour oncogene that promotes breast cancer cell proliferation, migration and invasion.

### 3.2 Effect of Nrf2 on pentose phosphate pathway in breast cancer cells

**Figure 3:**
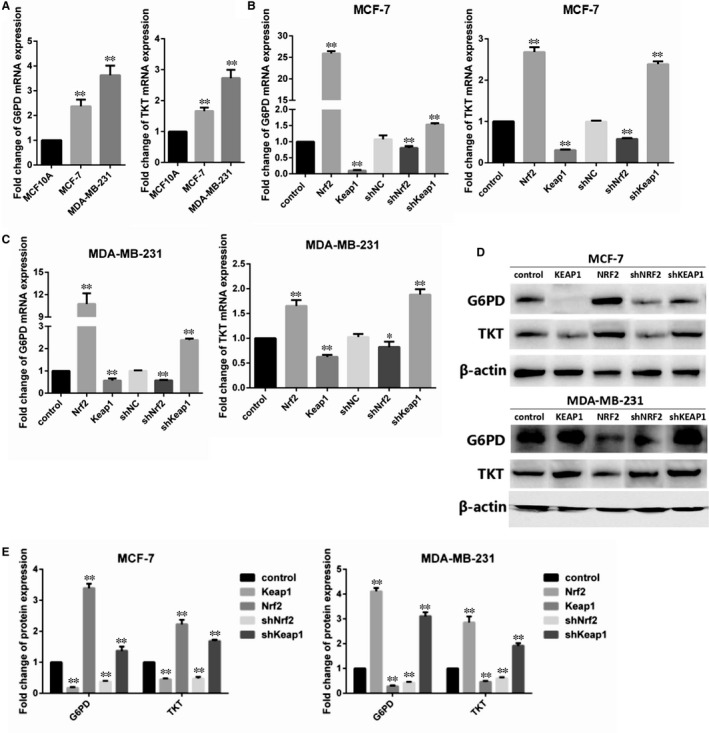
Effect of Nrf2 on pentose‐phosphate pathway in breast cancer cells. A, Expression of glucose‐6‐phosphate dehydrogenase (G6PD) and TKT mRNA in MCF‐10A, MCF‐7 and MBA‐DA‐231 cells was examined by qRT‐PCR. GADPH was used as a reference for RNA. (B) Expression of G6PD and TKT mRNA in MCF‐7 cells was examined by qRT‐PCR. GADPH was used as a reference for RNA. (C) Expression of G6PD and TKT mRNA in MBA‐DA‐231 cells was examined by qRT‐PCR. GADPH was used as a reference for RNA. (D) Expression of G6PD and TKT protein in MCF‐7 and MBA‐DA‐231 cells was performed by western blotting analysis. The signal obtained with a β‐actin antibody served as loading control. Blots were the representative of three independent experiments. (E) Quantitative analysis of G6PD and TKT protein in MCF‐7 and MBA‐DA‐231 cells was shown. Results were means ± SEM of three independent experiments. **P < 0.01 compared to untreated cells

Pentose phosphate pathway (PPP) is a major glucose metabolism pathway, which is fundamental in cancer growth and metastasis.^19^ To determine the expression of PPP in breast cancer cells, G6PD and TKT, the key enzymes of PPP were detected in MCF‐7 and MDA‐MB‐231 breast cancer cells as well as MCF‐10A control cells. We found that the key enzymes of PPP G6PD and TKT were increased in MCF‐7 and MDA‐MB‐231 cells compared with MCF‐10A cells (Figure 3A). In order to detect the effect of Nrf2 on PPP in breast cancer, expression of G6PD and TKT was detected by RT‐PCR and western blotting in MCF‐7 and MDA‐MB‐231 cells. The results were shown that Nrf2 overexpression and Keap1 knockdown increased the expression level of G6PD and TKT in MCF‐7 and MDA‐MB‐231 cells; while Keap1 overexpression and Nrf2 knockdown reduced the expression level of G6PD and TKT in MCF‐7 and MDA‐MB‐231 cells (Figure 3B‐D).

### 3.3 Nrf2 regulated Notch1 signalling in breast cancer cells

**Figure 4:**
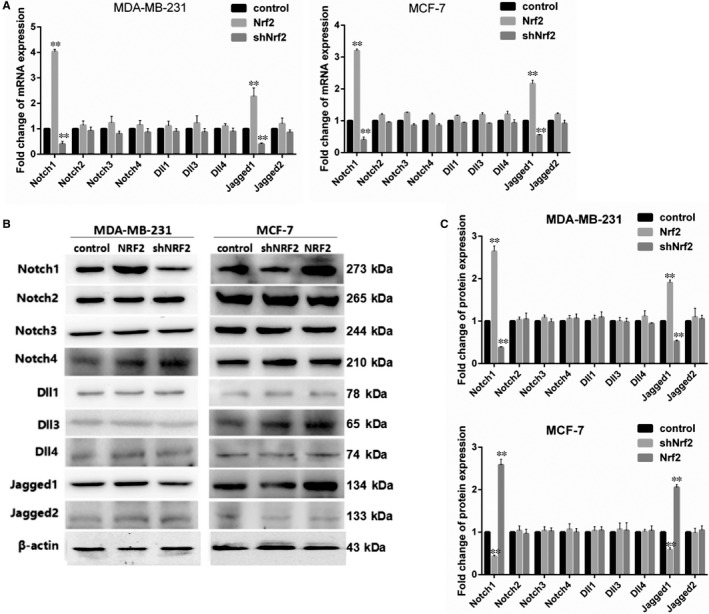
Nrf2 regulated Notch signalling in breast cancer cells. A, Expression of Notch1‐4, Dll1, Dll3, Dll4, Jagged1 and Jagged2 mRNA in MCF‐7 and MBA‐DA‐231 cells was examined by qRT‐PCR. GADPH was used as a reference for RNA. B, Expression of Notch1‐4, Dll1, Dll3, Dll4, Jagged1 and Jagged2 protein in MCF‐7 and MBA‐DA‐231 cells was performed by western blotting analysis. The signal obtained with a β‐actin antibody served as loading control. Blots were the representative of three independent experiments. C, Quantitative analysis of Notch1‐4, Dll1, Dll3, Dll4, Jagged1 and Jagged2 protein in MCF‐7 and MBA‐DA‐231 cells was shown. Results were means ± SEM of three independent experiments. **P < 0.01 compared to untreated cells

The main functions of Notch pathway are regulating cells proliferation and migration.^20^ In order to explore the influence of Nrf2 on Notch pathway in breast cancer cells, the expression of ligand and receptor of the Notch pathway were detected by RT‐PCR and western blotting in MCF‐7 and MDA‐MB‐231 cells. The results show high expression of Notch1 and Jagged1 in Nrf2 overexpression in MCF‐7 and MDA‐MB‐231 breast cancer cells; while Nrf2 knockdown in MCF‐7 and MDA‐MB‐231 cells had the opposite effect (Figure 4A,B). The expressions of Notch2‐4, Dll1, Dll3, Dll4 and Jagged 1‐2 were not affected by Nrf2 overexpression or Nrf2 knockdown in MCF‐7 and MDA‐MB‐231 cells (Figure 4A,B). It is suggested that Nrf2 is positively correlated with Notch1 in breast cancer.

### 3.4 Nrf2 regulated Notch1 signalling via G6PD/HIF‐1α in breast cancer cells

**Figure 5:**
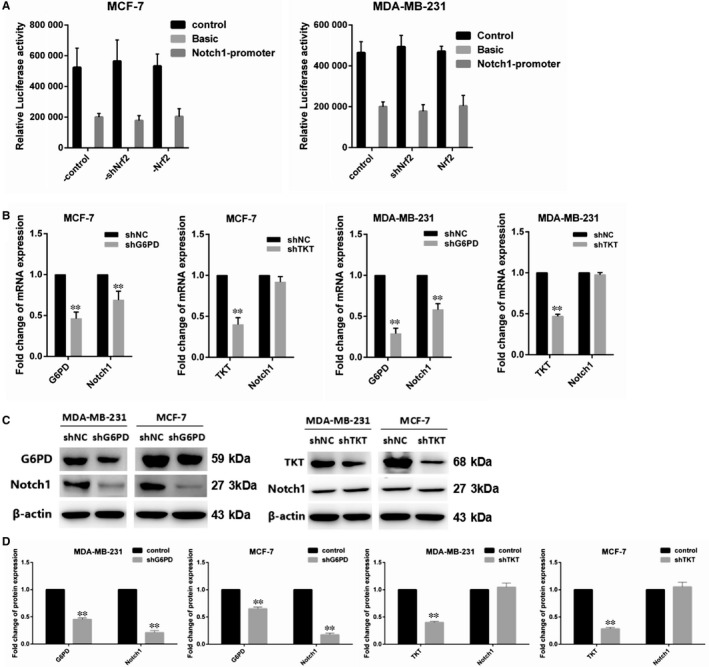
Nrf2 regulated Notch signalling via glucose‐6‐phosphate dehydrogenase (G6PD) in breast cancer cells. A, Relative luciferase activity was assayed in MCF‐7 and MBA‐DA‐231 cells. B, Expression of G6PD and Notch1 mRNA in MCF‐7 and MBA‐DA‐231 cells was examined by qRT‐PCR. GADPH was used as a reference for RNA. C, Expression of G6PD and Notch1 protein in MCF‐7 and MBA‐DA‐231 cells was performed by western blotting analysis. The signal obtained with a β‐actin antibody served as loading control. Blots were the representative of three independent experiments. D, Quantitative analysis of G6PD and Notch1 protein in MCF‐7 and MBA‐DA‐231 cells was shown. Results were means ± SEM of three independent experiments. **P < 0.01 compared to untreated cells

In order to determine whether Nrf2 directly activated Notch1 signalling pathway, Notch1‐promoter luciferase activity was assayed in MCF‐7 and MDA‐MB‐231 cells. The results show that Notch1‐promoter luciferase activity was not affected by Nrf2 overexpression or Nrf2 knockdown in MCF‐7 and MDA‐MB‐231 cells (Figure 5A). We assume that Nrf2 regulated Notch1 through PPP. The G6PD and TKT knockdown plasmid PLVX‐shG6PD and PLVX‐shTKT and non‐targeting plasmid PLVX‐shNC were built to transfection into MCF‐7 and MDA‐MB‐231 cells. The results show that mRNA and protein expression level of Notch1 was reduced in G6PD knockdown cells. But TKT knockdown had no effect on mRNA and protein expression of Notch1 (Figure 5B,C), which indicated that Nrf2 up‐regulated Notch1 expression via G6PD but not via TKT in breast cancer cells.

**Figure 6:**
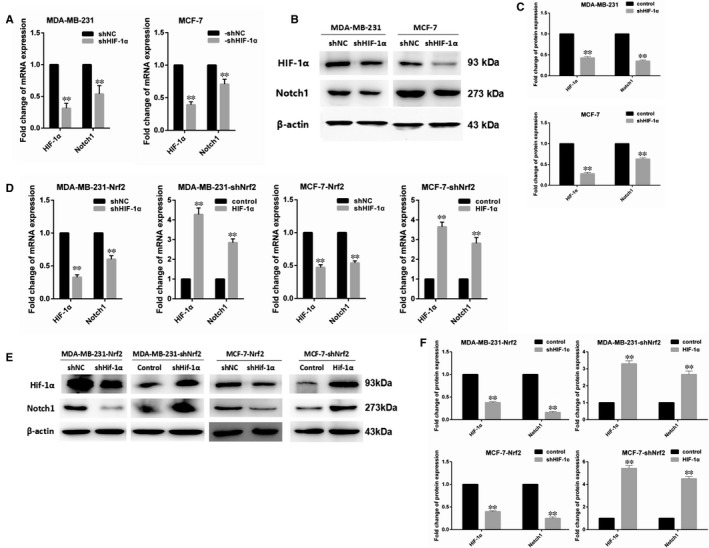
Nrf2 regulated Notch signalling via HIF‐1α in breast cancer cells. A, Expression of HIF‐1α and Notch1 mRNA in MCF‐7 and MBA‐DA‐231 cells was examined by qRT‐PCR. GADPH was used as a reference for RNA. B, Expression of HIF‐1α and Notch1 protein in MCF‐7 and MBA‐DA‐231 cells was performed by western blotting analysis. The signal obtained with a β‐actin antibody served as loading control. Blots were the representative of three independent experiments. C, Quantitative analysis of HIF‐1α and Notch1 protein in MCF‐7 and MBA‐DA‐231 cells was shown. D, Expression of HIF‐1α and Notch1 mRNA in Nrf2 overexpression or knockdown MCF‐7 and MBA‐DA‐231 cells was examined by qRT‐PCR. GADPH was used as a reference for RNA. E, Expression of HIF‐1α and Notch1 protein in Nrf2 overexpression or knockdown MCF‐7 and MBA‐DA‐231 cells was performed by western blotting analysis. The signal obtained with a β‐actin antibody served as loading control. Blots were the representative of three independent experiments. F, Quantitative analysis of HIF‐1α and Notch1 protein in Nrf2 overexpression or knockdown MCF‐7 and MBA‐DA‐231 cells was shown. Results were means ± SEM of three independent experiments. **P < 0.01 compared to untreated cells

One of the key goals of G‐6‐PD is to regulate the production of GSH and NADPH, and then regulate redox balance in vivo by varying the level of ROS. Hypoxia‐inducing factor 1α, which is sensitive to redox changes, is activated in hypoxia. We assumed that HIF‐1α influence the regulation of Notch1 by Nrf2. We investigated the expression of HIF‐1α in MCF‐7 and MDA‐MB‐231 cells using RT‐PCR and western blotting. The results demonstrate that mRNA and protein expressions of HIF‐1α were significantly increased in Nrf2 overexpression MCF‐7 and MDA‐MB‐231 cells; while the result of Nrf2 knockdown cells was not as expected (Figure 6A,B). Then HIF‐1α knockdown plasmid PLVX‐shHIF‐1α was constructed to transfect Nrf2 overexpression MCF‐7 and MDA‐MB‐231 cells. Both RT‐PCR and western blotting were implemented to detect the expression of HIF‐1α and Notch1. The results show that mRNA and protein expressions of Notch1 were decreased when reducing the expression of HIF‐1α in Nrf2 overexpression MCF‐7 and MDA‐MB‐231 cell; while mRNA and protein expressions of Notch1 were increased with overexpression of HIF‐1α in Nrf2 knockdown MCF‐7 and MDA‐MB‐231 cells (Figure 6C,D). It is suggested that Nrf2 regulated Notch1 by HIF‐1α‐mediated PPP change.

### 3.5 Nrf2 can affect breast cancer cell proliferation and migration by influencing the expression of Notch1

**Figure 7:**
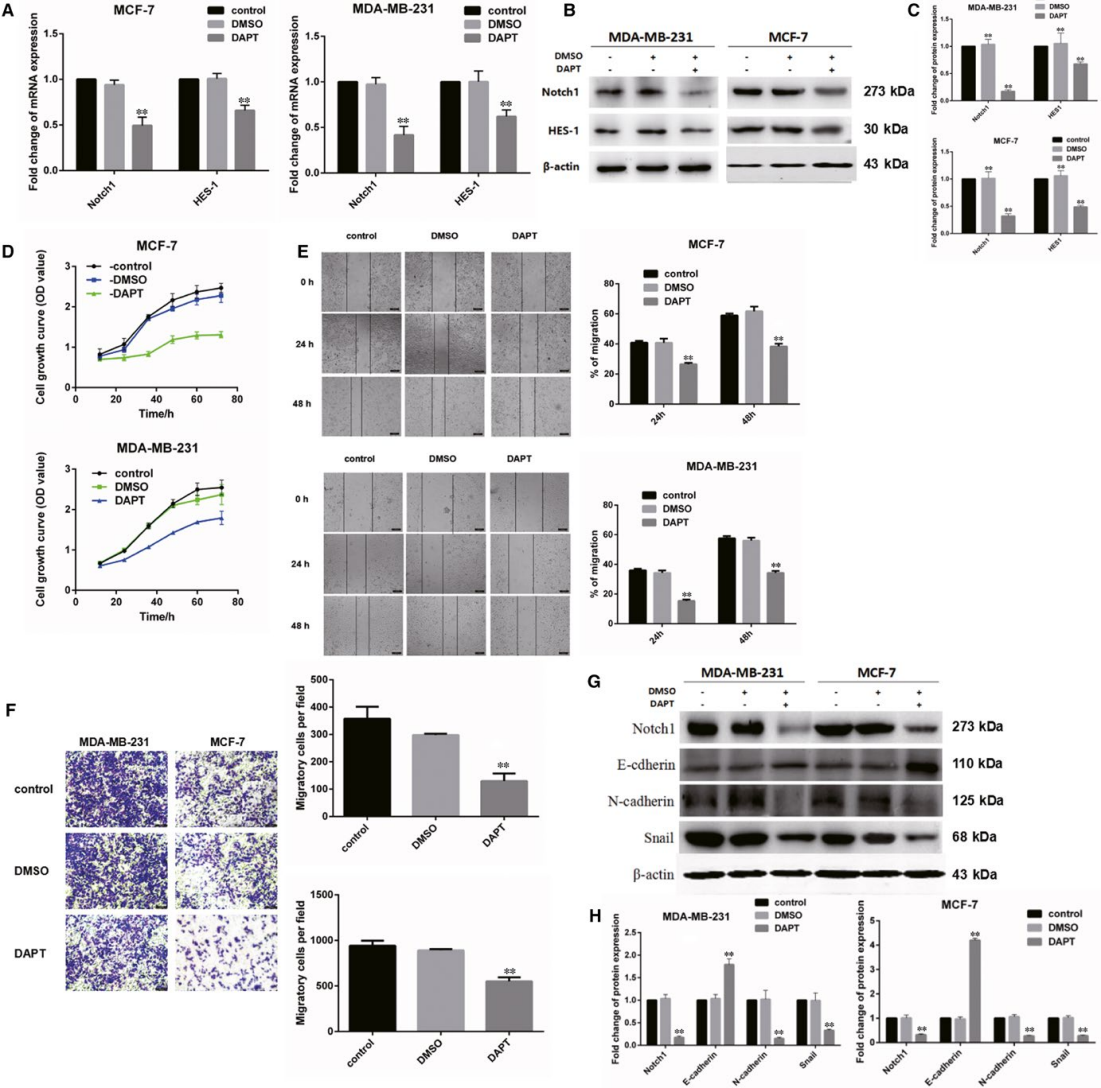
Nrf2 can affect breast cancer cell proliferation and migration by influencing the expression of Notch1. A, Expression of Notch1 and Hes1 mRNA in MCF‐7 and MBA‐DA‐231 cells was examined by qRT‐PCR. GADPH was used as a reference for RNA. B, Expression of Notch1 and Hes1 protein in MCF‐7 and MBA‐DA‐231 cells was performed by western blotting analysis. The signal obtained with a β‐actin antibody served as loading control. Blots were the representative of three independent experiments. C, Quantitative analysis of Notch1 and Hes1 protein in MCF‐7 and MBA‐DA‐231 cells was shown. D, Cell viability was analysed by MTS assay. E, Wound healing assay with MCF‐7 and MBA‐DA‐231 cells expressing shNrf2, shKeap1, Nrf2 or Kelch‐like ECH‐associated protein 1 (Keap1). Images were captured 24, 48, 72 h after wound was created. The percentage of migration was assigned as 100% when complete fusion occurred, and 0% at *t* = 0 h. Relative migratory rate was shown in the graph. F, Transwell invasion assay of MCF‐7 and MBA‐DA‐231 cells expressing shNrf2, shKeap1, Nrf2 or Keap1 for 24 h. Representative figures from three independent experiments were shown. G, Expression of Notch1, E‐cadherin, N‐cadherin and Snail1 protein in MCF‐7 and MBA‐DA‐231 cells was performed by western blotting analysis. The signal obtained with a β‐actin antibody served as loading control. Blots were the representative of three independent experiments. H, Quantitative analysis of Notch1, E‐cadherin, N‐cadherin and Snail1 protein in MCF‐7 and MBA‐DA‐231 cells was shown. Results were means ± SEM of three independent experiments. **P < 0.01 compared to untreated cells

One of the main functions of the Notch pathway involves regulating breast cancer cell proliferation and migration.^21^ We assumed that the effect of Nrf2 on breast cancer cell proliferation and migration was regulated through Notch pathway. The inhibitor of Notch signalling pathways (DAPT), a γ‐secretase inhibitor, was treated for 24 hours in MCF‐7 cells and MDA‐MB‐231 cells. Hairy and enhancer of the split homolog‐1 (HES‐1), as the downstream target genes of Notch1 protein, belong to the proneural basic helix‐loop‐helix (bHLH) gene family. The expression level of Notch1 and HES‐1 was detected through RT‐PCR and western blotting in MCF‐7 and MDA‐MB‐231 cells treated with DAPT. The results show that the expression of HES‐1 was reduced after inhibiting Notch pathway by DAPT (Figure 7A‐C). The results show that proliferation and migration of breast cancer cells MCF‐7 and MDA‐MB‐231 were decreased by DAPT (Figure 7D‐F). Therefore, we suggest that Nrf2 controls cell proliferation in breast cancer through influencing Notch1 and affecting HES‐1.

The invasion behaviour of malignant tumour is frequently associated with epithelial‐mesenchymal transition (EMT). We supposed that Nrf2 regulate cells migration in breast cancer by controlling Notch1 and changing EMT. Both RT‐PCR and western blotting were used to assay mRNA and protein level of Snail, E‐cadherin and N‐cadherin in cells that were handled with DAPT. The results show that the level of Snail and N‐cadherin was decreased, but the level of E‐cadherin was increased in DAPT‐treated MCF‐7 and MDA‐MB‐231 cells (Figure 7G,H).

## 4 DISCUSSION

Metabolic reprogramming by transcription factor Nrf2 could contribute to the development of breast cancer.^11^ However, the mechanisms that drive breast cancer progression need further research. The present results show that Nrf2 overexpression promoted tumour proliferation and migration in breast cancer cells MCF‐7 and MDA‐MB‐231, silencing of Nrf2 could suppress those cancer phenotype. Overexpression of Nrf2 or knockdown of Keap1 increased the expression of G6PD, the rate‐limiting enzyme of PPP. Overexpression of Nrf2 increased the expression of Notch1 gene via G6PD/HIF‐1α. The levels of HES‐1, Snail, E‐cadherin, N‐cadherin, the downstream gene of Notch1 pathway were changed by Notch1 up‐regulation. There were opposite effects through knockdown of Nrf2 gene or overexpression of Keap1. These findings suggest that Nrf2 was proved to be a novel therapeutic target to breast cancer, which provide a new intervention targets for intervention of breast cancer metastasis.

NF‐E2‐related factor 2 is important in protection of oxidative stress. However, some studies have shown that the survival and development of cancer cells were promoted through excessive expression of Nrf2 and its target genes.^22^ Recent data suggest that the there are different degrees of Keap1 mutations in patients with lung cancer.^23^ Activation of Nrf2 can promote cell tumourigenicity, and knockdown of Nrf2 can inhibit tumour growth, metastasis and invasion.^24^ The target genes of Nrf2 are vital in tumour metastasis. The main function of heme oxygenase 1 (HO‐1), the main target gene of Nrf2, is to participate in maintaining cells redox equilibrium. It is reported that the survival rate of lung cancer patients with high level of HO‐1 is lower than patients with low expression of HO‐1.^25^ NAD(P)H quinone dehydrogenase 1 (NQO1) is another gene activated by Nrf2 and also the key gene of oxidative stress. Compared with no‐metastatic tumours, NQO1 in metastatic tumours showed higher expression.^26^ In this study, we observed that high levels of Nrf2 expression significantly correlated with higher proliferation and migration of breast cancers cells, all of which precludes shorter overall survival and a higher recurrence rate in breast cancer patients. These observations suggest that Nrf2 promotes breast cancer metastasis and may serve as a biomarker for poor prognosis.

In addition to its role in regulation of oxidative stress, Nrf2 also involves in the anabolic metabolism. NF‐E2‐related factor 2 directly activates six genes involved in PPP and NADPH production pathway, including G6PD, PGD, TKT, TALDO1 and malic enzyme 1 (ME1), through binding of this transcription factor to AREs of these gene promoters.^11^ The metabolic reprogramming provides energy and metabolites to facilitate rapid growth and proliferation of cancer cells. The PPP is important in promoting cancer proliferation, differentiation and survival.^27^ Overexpression of G6PD is a prognostic factor in multiple cancers and is associated with tumour progression, metastasis or recurrent disease.^28^ The oxidative PPP converts G6P, a glycolytic intermediate, into ribulose‐5‐phosphate and generates NADPH, which is used for GSH production, detoxification and biosynthesis of lipids. Pentose phosphate pathway is up‐regulated in many types of tumours. The activities of G6PD and TKT, key PPP enzymes, were increased in cancer cells.^29^ In this work, we found that the two key PPP enzymes, G6PD and TKT, were up‐regulated in Nrf2‐overexpressed MCF‐7 and MDA‐MB‐231 cells, whereas ectopic overexpression of Keap1 and Nrf2 knockdown counteracts the increase of G6PD and TKT expressions in breast cancer cells. These observations indicate that PPP is activated in Nrf2‐overexpressed breast cancer cells. In addition, targeting Nrf2 as a combination therapy/chemically induced synthetic lethality target, an approach that has shown excellent results for breast cancer therapy.^30^, ^31^

**Figure 8:**
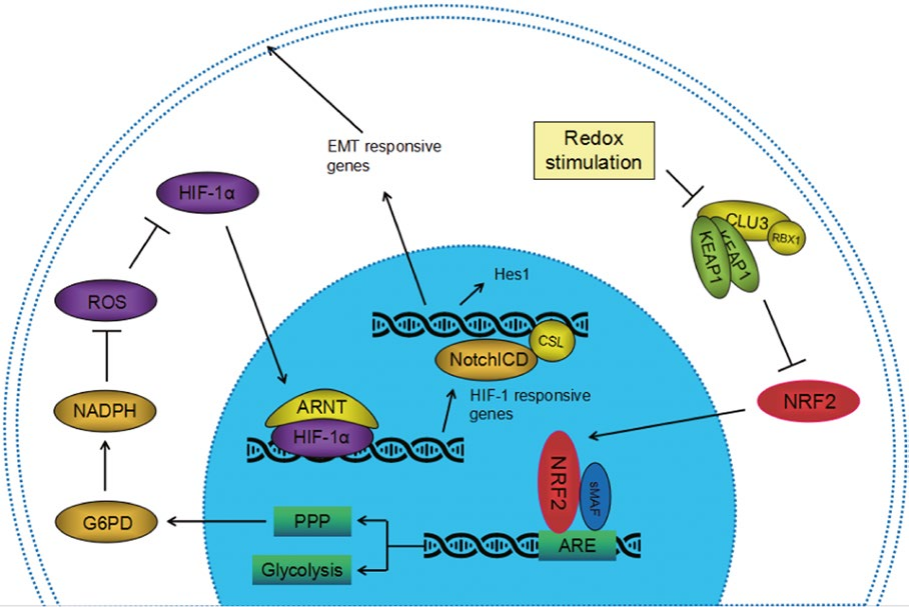
A model of Nrf2‐mediated Notch1 activation via glucose‐6‐phosphate dehydrogenase (G6PD)/HIF‐1α axis in breast cancer cells

NF‐E2‐related factor 2 can interact with other signalling pathways key genes. Notch pathway is one of them, Notch1 is crucial in cell survival, abnormal expression of Notch1 has been considered as a universal phenomenon in small cell lung cancer.^34^ NF‐E2‐related factor 2 can activate Notch1 and regulate the proliferation of haematopoietic progenitor cells (HPCs).^35^ The results are consistent with our findings that inhibition of Notch1 lowered the expression of its target genes HES‐1 and inhibited the tumour proliferation. The invasion behaviour of malignant tumour is accompanied with loss of E‐cadherin, and increase of N‐cadherin. Cut Notch1 can reduce EMT pathway. Previous research has shown that Notch1 downstream gene expression is related to the EMT. This is in agreement with the results of our research that inhibition of Notch1 enhances the expression of E‐cadherin, but lowers the expression of Snail and N‐cadherin. These results indicate the reduction of EMT pathway by Notch in breast cancer cells and the importance of Notch1 in breast cancer cells. A model of Nrf2‐mediated Notch1 activation via G6PD/HIF‐1α axis in breast cancer cells has been shown in Figure 8.

Our conclusion confirmed the involvement of Nrf2 transcription factor in tumour metastasis. In summary, our findings suggest that Nrf2 contributes to metastatic ability of basal type breast cancer cells through G6PD/HIF‐1α/Notch1 signalling axis. Nrf2‐dependent G6PD/HIF‐1α activation provokes Notch1 signalling by up‐regulation of Jagged1 and Hes1, thereby promoting EMT in breast cancer cells. Further study on Nrf2/G6PD/HIF‐1α/Notch1 signalling axis is demanded for realization of basal type breast cancer treatment.

## CONFLICT OF INTEREST

The authors declare that there are no conflicts of interest.
